# Reduced Th1 and Treg immune responses in a pediatric egg-allergic group susceptible for an OIT protocol

**DOI:** 10.1186/2045-7022-3-S3-P49

**Published:** 2013-07-25

**Authors:** I López Expósito, M Reche, T Valbuena, A Padial, C Pascual, L Perezábad, R López-Fandiño, E Molina

**Affiliations:** 1 Bioactivity and Food Analysis, Institute for Food Science Research (CIAL) (CSIC-UAM), Madrid, Spain; 2 Allergy Service, Infanta Sofia Hospital, Madrid, Spain

## Background

Egg allergy is one of the most common food allergies of childhood with an estimated prevalence of 2.6 %. Allergic reactions to egg may vary from atopic dermatitis to systemic anaphylaxis. Although traditionally the only treatment is dietary avoidance, oral immunotherapy (OIT) appears as a promising approach to induce egg-tolerance. However, the immune mechanisms underlying this treatment are largely unknown. The purpose of this study was to compare the clinical and immune responses against egg allergens of a pediatric population candidate for an OIT protocol with a non-atopic population of the same age range.

## Methods

A group of 19 pediatric patients with egg allergy confirmed by egg-specific IgE levels and positive double blind placebo controlled tests (DBPCT) was compared with a group of 9 non-atopic children on an egg-containing diet. The mean age of the egg-allergic patients was 10.1 years compared with 6.2 years in the control group. No significant differences regarding sex or age were found between the two groups. In both groups, PBMCs were isolated from peripheral blood and stimulated during 7 days with 200 μg/mL of ovalbumin (OVA). Culture supernatants were analyzed for IL-5, IL-13, IL-1β, IFN-γ, TNF-α and IL-10 production by Cytometric Bead Array (CBA).

## Results

During the DBPCT with pasteurized egg-white, 8 egg-allergic patients had urticaria, 5 anaphylaxis, 4 experienced digestive symptoms and 2 respiratory symptoms. The mean positive dose in the DBPCT was 1.4 mL of egg-white, within a range of 0.001-8 mL. Specific IgE measurements revealed, on average, 71.0 kU/L for egg-white, 31.7 kU/L for OVA and 45.6 kU/L for ovomucoid.

CBA results showed a significantly impaired production of OVA-specific IFN-γ (P<0.05), TNF-α (P<0.01) and IL-10 (P<0.01) in egg-allergic patients when compared with control children. In addition, a trend toward higher OVA-specific IL-5 and IL-13 production was found in egg-allergic patients.

## Conclusion

Under the conditions studied, egg-allergic children showed a significantly reduced Th1 and Treg cytokine production upon stimulation with OVA in comparison with non-atopic controls. Furthermore, a trend toward the production of IL-5 and IL-13 was observed. The clinical response to egg in the allergic group was reflected in the T-cell responses to OVA and the evolution of the cytokine profile upon a OIT protocol might provide a better understanding of the immune responses involved in allergy and tolerance to egg.

## Disclosure of interest

None declared.

